# The Impact of University Innovation and Entrepreneurship Education on Entrepreneurial Intention From the Perspective of Educational Psychology

**DOI:** 10.3389/fpsyg.2021.745976

**Published:** 2021-11-30

**Authors:** Qi Wang, Zhiwei Sun, Cisheng Wu

**Affiliations:** ^1^School of Management, Hefei University of Technology, Hefei, China; ^2^Faculty of Mathematics and Statistics, Suzhou University, Suzhou, China

**Keywords:** educational psychology, innovation and entrepreneurship education, entrepreneurial knowledge, entrepreneurial intention, mediation model

## Abstract

In recent years, innovation and entrepreneurship education is one of the hot spots of higher education research and practice in China. Innovation and entrepreneurship education can be taken as a new direction. Despite the commonly held view that entrepreneurship education drive the promotion of entrepreneurial intention, little is known about the mechanism through which this intervention impacts on entrepreneurial intention. Based on the assimilation learning theory of cognitive structure and the planned behavior theory, the purpose of this work is to analyze the relationship between innovation and entrepreneurship education (IEE) and entrepreneurial intention from the perspective of educational psychology, to explore the influence mechanism of IEE on entrepreneurial intention, and to promote the success of college students in entrepreneurship. In this study, questionnaires were conducted among college students of different colleges, majors, and grades in Anhui province, with the aid of multiple linear regression analysis and mediating effect model analysis. The results show that IEE has positive effect on entrepreneurial intention. In addition, entrepreneurial knowledge plays a mediating role in the relationship between IEE and entrepreneurial intention. And the mediating effect of innovation behavior and entrepreneurial attitude is not obvious. These results are expected to provide evidence for understanding the relationship between IEE and entrepreneurial intention under the background of Chinese education, to enrich and expand the theoretical basis of IEE, and to possess theoretical, empirical, and practical significance for the design and implementation of innovation and entrepreneurship education for college students.

## Introduction

In recent years, innovation and entrepreneurship education (IEE) has been a hot topic in the academic community. Since 2016, innovation and entrepreneurship as the core qualities of talent development have grown up. The concept of IEE originated from Harvard Business School, and later, a course on entrepreneurship was opened in Harvard Business School in 1947. After that, IEE in universities was rapidly popularized all over the world (Nabi et al., 2017). The purpose of IEE is to cultivate innovative talents, namely, students who will be able to challenge the status quo with a pioneering spirit, adventure, and a sense of independence and to solve problems creatively to meet demands of the society (Fayolle, 2013). Ferreira and other scholars emphasize the important role of entrepreneurship education in promoting entrepreneurship intention and entrepreneurial behavior (Ferreira and Pinheiro, 2018). García-Rodríguez et al. (2017) have shown that entrepreneurship education is effective in influencing students’ entrepreneurial attitudes and intentions (Anggadwita et al., 2017; García-Rodríguez et al., 2017). Besides, researches showed that the innovation and entrepreneurship courses improve entrepreneurial attitude and intention of students. (Gielnik et al., 2015; Muscio et al., 2019).

Bird first proposed the concept of entrepreneurial intention and believed that entrepreneurship was a kind of willing and planned behavior (Bird, 1988). Entrepreneurial intention reflects the motivation of individuals to put conscious plans or decisions into action. Bagheri thought that entrepreneurial intention referred to a subjective attitude taken by potential entrepreneurs toward the decision of starting a business, to measure the degree of entrepreneurial traits (Bagheri and Pihie, 2015). Entrepreneurial intention is only a motivation, which may not lead to the occurrence of entrepreneurial behavior (Salamzadeh et al., 2013). However, entrepreneurial intention is the prerequisite for entrepreneurial behavior, and those who have started businesses in the past must take entrepreneurial intention as the guide (Qiao and Huang, 2019). Many researchers indicated that there is a positive relationship between entrepreneurial education and entrepreneurial intention (Kariv et al., 2019; Zhang et al., 2020). Another study also confirmed that entrepreneurship education encourages students to pursue entrepreneurship as a career and gives students the skills they need to start a business (Chen, 2019; Marlous et al., 2021).

The purpose of this paper mainly includes the following three aspects:

1. The first purpose of this paper is to explore the impact of IEE on college students’ entrepreneurial intention, based on the assimilation learning theory of cognitive structure and planned behavior theory. At present, there are many discussions about the impact of IEE on entrepreneurial intention, but the conclusions are quite different. Some studies believe that IEE can promote entrepreneurial intention of students, but some research suggest that the effect of IEE on students’ entrepreneurial intention is insignificant or even negative. The conclusions lack generality. This shows that the specific role of IEE has not been fully explored.
2. The second purpose of this paper is to examine the mediating effect of entrepreneurial attitude, innovative behavior and entrepreneurial knowledge on the relationship between IEE and entrepreneurial intention of students. So, is there a certain logical relationship between IEE and entrepreneurial intention? Can IEE effectively improve entrepreneurial intention of students? If there is a logical relationship between them, furthermore, what is the mechanism of action and how does it work? Therefore, the impact of IEE on entrepreneurial intention needs to be verified. As a consequence, the article attempts to prove that the impact of IEE on entrepreneurial intention has a positive and statistical relationship with specific innovation behavior, innovation knowledge, and entrepreneurial attitude.
3. The third aim of this study is to enrich and expand the theoretical basis of IEE and to offer constructive suggestions for design and implementation of IEE. Previous studies show that education is an important way to train entrepreneurial intention and innovative ability (Martin et al., 2013). This view has led to a large amount of global investment in IEE, such as the dramatic increase in the number of innovation and entrepreneurship courses in universities. So, how education can maximize the promotion of college students’ innovative character and entrepreneurial consciousness. This article tries to give objective and reasonable suggestions after empirical testing.

## Theoretical Foundation

### Cognitive Assimilation Theory of Learning

Ausubel, a famous American cognitive educational psychologist, proposed a unique theory of “meaningful learning,” namely, “cognitive assimilation theory.” The theory belongs to the category of educational psychology theory, as an important part of educational psychology (Ausubel et al., 1978). Zhijie Tian consider that the purpose of meaningful learning is to establish a connection between old and new knowledge to obtain the psychological meaning of knowledge. Therefore, students need to take the initiative: (1) to “judge” the adaptability between old and new knowledge; (2) to “adjust” the differences and contradictions between old and new knowledge; (3) to “link” the background and experience between old and new knowledge; and (4) to “reorganize” general and inclusive contents to link the old and new knowledge in a higher form (Tian et al., 2020). Interestingly, these four procedures are related to the process of students’ thinking. According to this theory, meaningful learning will enable students to effectively accumulate entrepreneurial knowledge and then make students familiar with entrepreneurial scenes, so as to generate a strong entrepreneurial will. This provides a new theoretical perspective for this study to explore the relationship between innovation and entrepreneurship education and entrepreneurial intention.

### Theory of Planned Behavior

**Figure 1:**
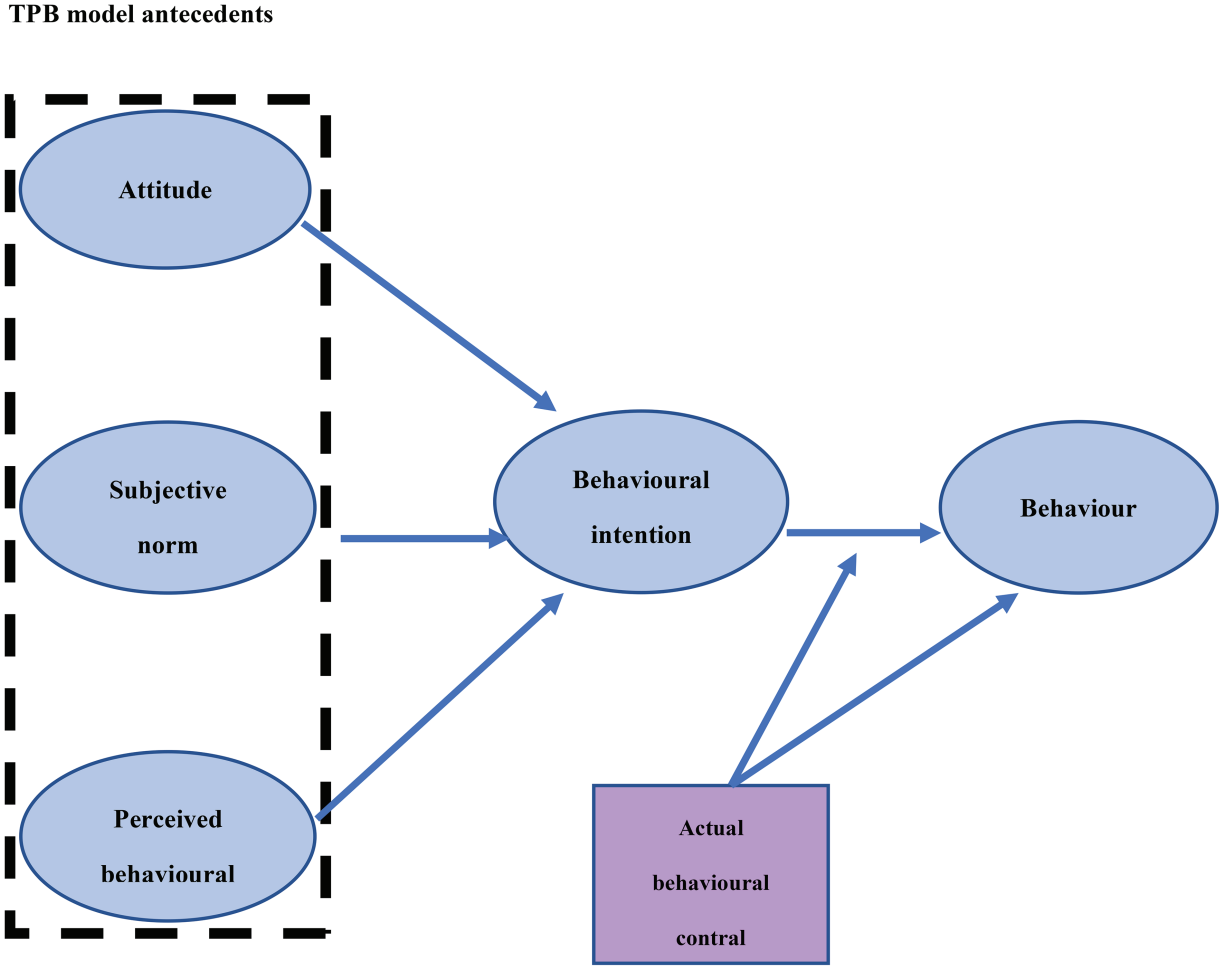
Overview of the theory of planned behavior.

Psychologist Ajzen proposed the theory of planned behavior in Late 20th century, which evolved from theory of reasoned action (Ajzen, 1991). According to Ajzen, Individuals’ behavioral beliefs have both positive and negative effects on their behavioral attitudes. Normative beliefs produce perceived social pressure or subjective norms, while control beliefs have a decisive effect on perceived behavioral control (Ajzen, 2002; Zhong et al., 2020; As shown in Figure 1). García-Rodríguez considers that intention is an important factor in identifying someone’s motivations and characteristics in establishing entrepreneurial activities (García-Rodríguez et al., 2017). The willingness to perform different behaviors can be predicted through behavioral attitudes, subjective norms, and perceived behavioral control, which have an important impact on the actual behavior. Therefore, the more positive the behavioral attitude and subjective norms are, the stronger the perceived behavioral control the individual’s willingness to perform a certain behavior will be. Its specific mechanism is shown in Figure 1. Since the advent of the theory of planned behavior, many scholars at home and abroad have used the theory of planned behavior to conduct empirical tests in the field of mass entrepreneurship and innovation research. The research results have shown that this model has a good prediction effect (Shen et al., 2019).

## Literature Review and Research Hypothesis

### IEE and Entrepreneurial Intention

IEE refers to the collection of all forms of knowledge transfer of innovation and entrepreneurship (Bird, 1988). Bird further proposed that entrepreneurial intention was a kind of mental state that promoted individuals to form new business concepts (Bird, 1988). More attention has paid to the relationship between IEE and entrepreneurial intention. Anjum et al. (2018) found that IEE can enhance feasibility of perception of students by increasing their knowledge. As a result, entrepreneurial intention of students was enhanced (Anjum et al., 2018). Martin found that innovation and entrepreneurship education can not only improve entrepreneurial knowledge and skills of students, but also significantly enhance entrepreneurial intention of students (Martin et al., 2013). Rideout and Gray comment that there is a close relationship between entrepreneurial education and entrepreneurial intention (Rideout and Gray, 2013). On the basis of the existing studies, we propose the following hypothesis in this work:

*H1*: IEE has a positive impact on entrepreneurial intention.

### Entrepreneurial Knowledge and Entrepreneurial Intention

Hamilton considered that entrepreneurial learning can help students acquire, accumulate, and create entrepreneurial knowledge (Hamilton, 2011). According to Volery, entrepreneurship education can help students accumulate useful knowledge of entrepreneurship, thus forming individual human capital (Volery et al., 2013). IEE conveys entrepreneurial knowledge to students. By practical trainings, IEE provides students the opportunity to experience and imperceptibly improving students’ sense of identity for entrepreneurship. Once students have acquired basic entrepreneurial knowledge and skills, they will subconsciously reevaluate the possibility of starting a business according to their actual conditions. Therefore, entrepreneurial intention of students will be improved. So, the authors believe that entrepreneurial knowledge can be acquired through meaningful learning and that the accumulation of entrepreneurial knowledge is helpful to stimulating college students’ entrepreneurial intention. Based on the above discussion, the following hypothesis is proposed:

*H2*: Entrepreneurial knowledge has a positive impact on entrepreneurial intention.

### Innovative Behavior and Entrepreneurial Intention

West and Farr defined innovative behavior as an activity in which individuals generate, introduce, and apply new ideas and procedures to the organization (West and Farr, 1989). Some scholars also defined innovative behavior as a multi-stage process consisting of the generation, promotion, and practice of ideas (Scott and Bruce, 1994). Since college students are still in their learning stage, innovation behavior can be regarded as a dynamic process put their new ideas into practice under certain circumstances and actively promote the improvement of team performance. Pihie thought that individual entrepreneurial intention is closely related to potential ability (Pihie and Bagheri, 2013; Feng and Chen, 2020). Therefore, we propose the following hypothesis:

*H3*: Innovative behavior has a positive impact on entrepreneurial intention.

### Entrepreneurial Attitude and Entrepreneurial Intention

In the field of psychology, attitude has been proved to be effective predictors of behavioral intention. Pandit considered that the individual’s attitude toward a certain behavior will significantly affect the behavioral intention (Pandit et al., 2018; Li et al., 2019). The more positive an individual’s evaluation of the results of entrepreneurship is, the more positive whose attitude toward entrepreneurship will be, and accordingly, the stronger his willingness to start a business will be. Luthje and Franke discussed the correlation between college students’ entrepreneurial attitude and entrepreneurial intention. Linan and Chen took college students in Spain and Taiwan as the survey subjects to study entrepreneurial intention, respectively (Linan and Chen, 2009). The results showed that the entrepreneurial attitude of college students in the two regions had a significant impact on entrepreneurial intention. Based on existing studies, the following hypothesis is proposed in this article:

*H4*: Entrepreneurial attitude has a positive impact on entrepreneurial intention.

### IEE and Entrepreneurial Knowledge

IEE has a demonstration role, which can arouse learning desire and instinct of imitation of people. Hejazinia (2015) believe that IEE is one of the important factors affecting college students’ willingness to start businesses. Duval-Couetil et al. (2021) found in their study that the possibility of students choosing to start their own businesses will be improved accordingly with the correct demonstration and material support for students in entrepreneurship and innovation education (Duval-Couetil et al., 2021). According to Kamil and Nasurdin, entrepreneurial behavior is a series of processes in which entrepreneurs experience entrepreneurial opportunity discovery, entrepreneurial opportunity evaluation, and entrepreneurial action (Baluku et al., 2019). Ausubel argued that as long as one strives to influence the cognitive structure to enhance meaningful learning, the core of the educational process can be elucidated (Ausubel et al., 1978). In the process of IEE, the learning of entrepreneurship knowledge is also a way for students to connect the knowledge of innovation and entrepreneurship with their own cognitive structure to conduct meaningful learning. According to the cognitive assimilation theory, we put forward the following hypothesis:

*H5*: IEE has a positive impact on entrepreneurial knowledge.

### IEE and Innovative Behavior

Scott and Bruce (1994) believed that innovative behavior is a process of recognizing problems, generating ideas, and then putting ideas into practice by seeking support and help. Some scholars believe that innovative behavior refers to the behavior that employees generate new ideas and embody new ideas into various innovative activities. Others believe that individual innovation behavior should be a complex combination of new ideas generation, introduction, realization, or fulfillment (Martin and Widjaja, 2019). At present, it is shown that innovation behavior is a behavior outside the core task performance, including the three stages of generation of new ideas, promoting the dissemination of ideas, and the implementation of ideas (Thomas and Daniel, 2013). In the process of teaching or training students, innovation and entrepreneurship education will naturally make students generate new ideas based on the knowledge they have acquired and promote the dissemination and realization of ideas. Therefore, the authors propose the following hypothesis:

*H6*: IEE has a positive impact on Innovative behavior.

### IEE and Entrepreneurial Intention

Entrepreneurial attitude is the degree of people’s views and preferences on entrepreneurial activities, as well as the degree of cognition of the impact of participation in entrepreneurial activities. The attitude and cognition of a person toward entrepreneurial activities will directly affect his or her willingness to participate in this activity and eventually have an indirect impact on behavior (Salamzadeh et al., 2014). Jonathan studied the influence of college students’ personal traits on their attitude toward entrepreneurship from the perspective of the content of entrepreneurship education (Jonathan and Linton, 2021). The results found that IEE can improve college students’ attitude toward entrepreneurship. According to the theory of planned behavior proposed by Ajzen (2002), any individual’s specific behavior is affected by behavioral intention, which in turn depends on the individual’s attitude and subjective norms toward the behavior (Palalic et al., 2016). Karimi believe that individual entrepreneurial attitude may be influenced by entrepreneurial education and previous entrepreneurial experience (Yu et al., 2020). Based on previous literature, this paper proposes the following hypothesis.

*H7*: IEE has a positive impact on entrepreneurial intention.

### The Mediating Role of Entrepreneurial Attitude

Drawing form Maha understanding about attitude of innovation, the author define the innovative attitude of college students as follows: under certain circumstances, college students’ cognitive, emotional and behavioral tendencies toward innovative ideas, innovative tasks, and innovative behaviors can be understood as the degree of control over innovative behaviors after considering the interaction between the individual and the learning environment (Maha et al., 2021). According to the theory of planned behavior, the intention of a person will influence his or her subsequent behavior, and attitude is an important factor to determine whether an individual has the intention or not. Luthje and Franke took science and engineering students as the research object and found that their entrepreneurial intention was significantly affected by their entrepreneurial attitude (Luthje and Franke, 2004). Therefore, the authors propose the following hypothesis.

*H8*: Entrepreneurial attitude plays a mediating role in the relationship between IEE and entrepreneurial intention.

### The Mediating Role of Innovation Behavior

It can be seen that there may be a logical relationship among Innovation and entrepreneurial education, innovative behavior, and entrepreneurial intention. Through innovation and entrepreneurship education, students will acquire knowledge of innovation and entrepreneurship and obtain the ability and characteristics conducive to entrepreneurship. These students will be likely to engage in innovative activities, such as participating in entrepreneurship competition, publishing academic papers, publishing invention patents, trying to establish a new company, and so on. The realization of the innovative behavior of the students will stimulate their own entrepreneurial intention (Neneh, 2019; Deng et al., 2021). Therefore, based on the above discussion, the authors proposes a hypothesis. H9: Innovation behavior plays a mediating role in the relationship between IEE and entrepreneurial intention.

*H9*: Innovation behavior plays a mediating role in the relationship between IEE and entrepreneurial intention.

### The Mediating Role of Entrepreneurial Knowledge

According to Hamilton (2011), entrepreneurial learning can help students acquire, accumulate, and create entrepreneurial knowledge. Some scholars have explained it from the perspective of human capital. Volery believed that entrepreneurship education can accumulate useful entrepreneurial knowledge for the students, thus forming individual human capital (Volery et al., 2013). The general consensus in academic circles is that the entrepreneurial ability of individuals is not innate, but acquired through learning and cultivation. Academics explored the internal mechanism of the transformation of entrepreneurs’ experience into entrepreneurial ability from the perspective of entrepreneurial learning and believed that the process of the transformation of entrepreneurs’ experience into entrepreneurial ability is a learning process. The main content of entrepreneurship education is the knowledge and ability of entrepreneurship. Based on the above discussion, the authors put forward a hypothesis.

*H10*: Entrepreneurial knowledge plays a mediating role in the relationship between IEE and entrepreneurial intention.

**Figure 2:**
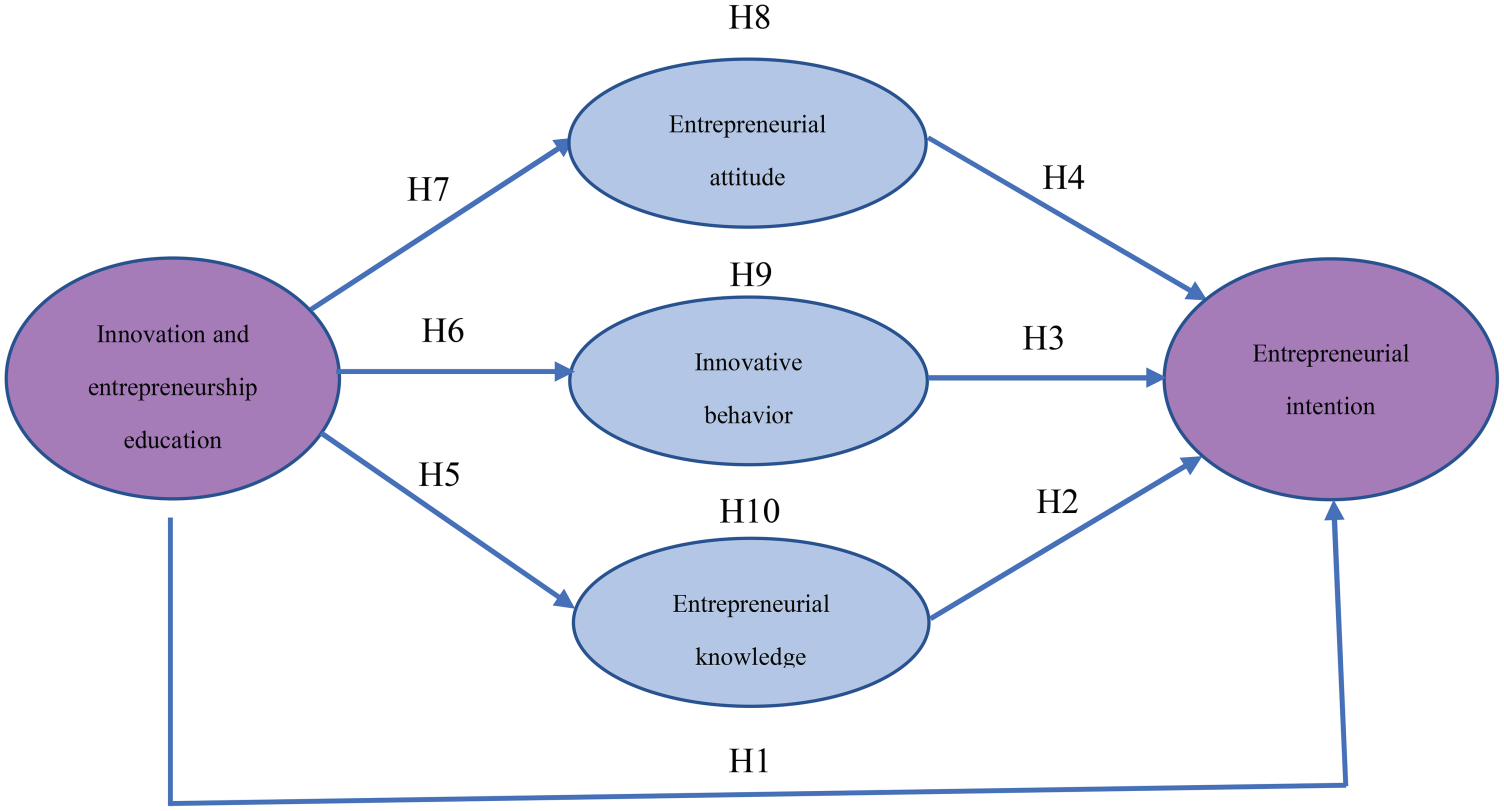
The mechanism model of entrepreneurial education.

To sum up, the hypotheses and theoretical model of this paper are shown in Figure 2.

## Research Methods

### Sample and Data Collection

This study takes college students as the research object. Before the formal distribution of the questionnaire, this study invited 5 experts in the field of entrepreneurship education to review the questionnaire and selected two universities in Anhui Province for pre-investigation, so as to adjust and revise the questionnaire. The research group carried out the survey in a number of universities in Anhui province by distributing the electronic questionnaire online. A total of 500 questionnaires were sent out, and 460 were received with a recovery rate of 92%. After eliminating the invalid questionnaires, a total of 414 valid questionnaires were collected with an effective recovery of 82.8%. The distribution of sample composition is shown in Table 1.

**Table 1:** Distribution of samples (*N*=414).

Variables	Classification	Number of the samples	Proportion (%)
Gender	Males Females	245	59.18
		169	40.82
Major	Liberal arts Economics Management Pedagogy Science and Engineering Other subjects	13	3.14
		91	21.98
		59	14.25
		233	56.28
		18	4.35
Grade	Freshman Sophomore Junior Senior	269	64.98
		59	14.25
		43	10.39
		43	10.39
Educational level	Junior college Bachelor degree Master’s degree Doctor’s degree	35	8.45
		327	78.99
		44	10.63
		8	1.93

### Variables and Measurements

In order to ensure the reliability and validity of the measurement, we adopt existing scale developed as a tool to collect empirical data, which is appropriately modified according to the purpose of this study. Likert’s five-point scale was used to score the questionnaire. The scale of innovation and entrepreneurship education draws on the research of Franke and Lüthje; meanwhile, we follow in Min Zhang’s footsteps for the entrepreneurial knowledge scale; besides, Ajzen afforded us lessons for the entrepreneurial attitude scale. The innovative behavior scale is based on the research of Scott and Bruce; as to entrepreneurial Intention Scale, we co-opted many from the research of Linán and Chen. The items are summarized in Table 2. In order to avoid the bias to the greatest extent, this study also measured some basic personal information, such as gender, major, grade, and education level, and took it as a control variable to improve the reliability of the research results.

**Table 2:** The measurement item of each variable.

Variable	Measurement items	Item number
IEE	The innovation and entrepreneurship education in the school has increased my interest in entrepreneurship	6(X1–X6)
	The innovation and entrepreneurship education in the school enables me to master business knowledge better	
	The innovation and entrepreneurship education in the school has improved my understanding of business opportunities	
	The innovation and entrepreneurship education in the school has improved my business management skills	
	The school has a sound entrepreneurial base or guidance institutions	
	The school has excellent guidance teachers for innovation and entrepreneurship	
Entrepreneurial knowledge	Enhance your understanding of entrepreneurs’ qualities, motivations and other aspects	5(X7–X11)
	Increase your understanding of the basic activities required to start a business	
	Increase your skills in entrepreneurial practice	
	Improve your ability to identify opportunities	
	Increase your understanding of the relationships needed to start a business	
Entrepreneurial attitude	I like to enjoy the challenge	5(X12–X16)
	I hope to achieve personal success	
	I wish to acquire more money and wealth	
	I want to be recognized by society	
	I hope to make my own contribution to the country’s economic development	
Innovation behavior	I often come up with some innovative ideas	5(X17–X21)
	I will introduce my new ideas to people around me to gain support and recognition	
	I try to get the resources I need to implement my idea or conception	
	I take the initiative to make plans to implement my innovative ideas	
	I often participate in discussions and contribute ideas to other people’s innovative ideas	
Entrepreneurial intention	I think I will start my own business in the future	5(Y1–Y5)
	I once considered self-employment	
	If given the chance, I will choose to start my own business	
	I will try my best to succeed in my own business	
	I think it’s very likely to start my own business in the next five years	

## Analysis of Empirical Results

### Reliability and Validity Analysis

This study takes Cronbach ă to measure the reliability of the scale and use the SPSS25.0 to test the reliability of the variables. The values of a Sig are all 0.000, which indicate that the data can be analyzed by factor analysis. Table 3 depicts the following results. Cronbach coefficients of all variables are higher than 0.7, and the Kaiser-Meyer-Olkin of all variables are greater than 0.7, indicating that the questionnaire had a high validity. The cumulative variance interpretation rate of each variable is more than 75%, indicating that the questionnaire designed has very good structural validity. It can be seen that the scale selected in this study not only has high overall reliability, but also has good internal consistency.

**Table 3:** Reliability and validity analysis results.

Variables	Cronbach ǎ	CFA	KMO	Sig.	CVI
IEE	0.953	0.881	0.909	0.000	81.159%
Entrepreneurial knowledge	0.963	0.927	0.901	0.000	87.112%
Entrepreneurial attitude	0.925	0.793	0.873	0.000	77.957%
Innovation behavior	0.946	0.887	0.911	0.000	82.234%
Entrepreneurial intention	0.931	0.863	0.891	0.000	78.632%

CFA, Confirmatory factor analysis, refers to the minimum factor loading in this paper.

**Figure 3:**
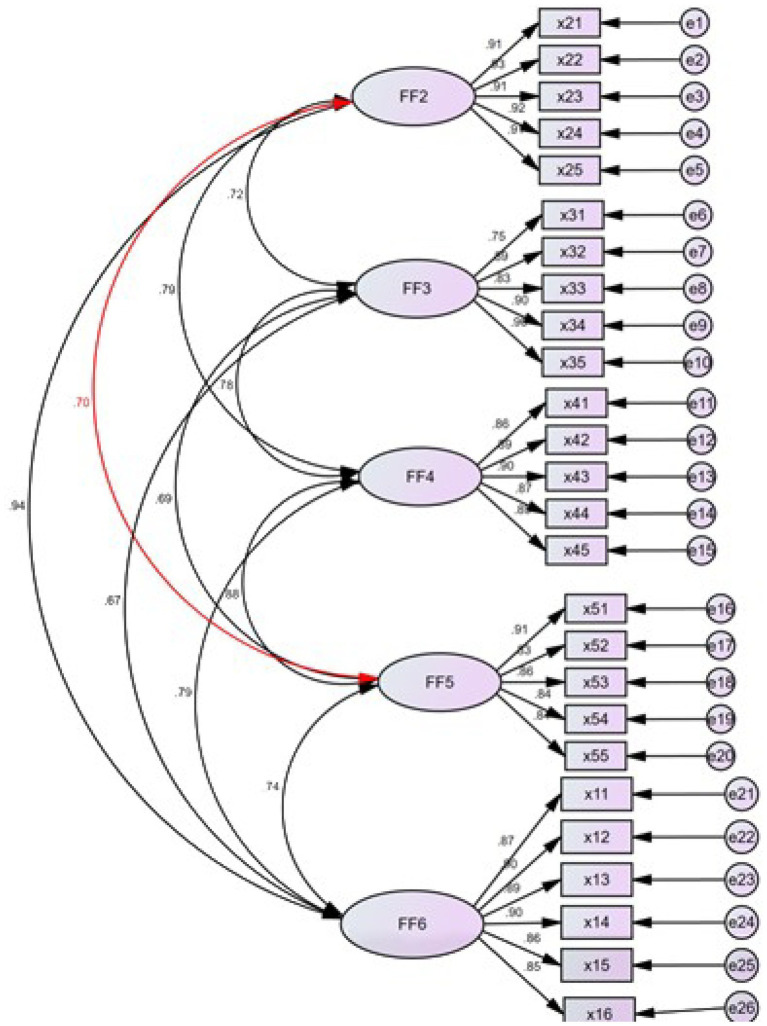
The structural equation path analysis of the model.

According to the theoretical research model proposed before, this paper constructs the structural equation model. With the help of AMOS23.0, a path graph between variables is drawn. As shown in Figure 3, for the sake of convenience, we used F2-F6 to represent the variables. Innovation and entrepreneurship education are represented by F2, entrepreneurial knowledge is represented by F3, entrepreneurial attitude is represented by F4, innovation behavior is represented by F5, and entrepreneurial intention is represented by F6. In Figure 3, every latent variable is represented by ellipses and each measurement item is represented by a rectangle. In the whole structure model, there are 5 latent variables and 26 manifest variables. In addition, there are 26 residual variables. The standardized path coefficients of each set of variables are all >0.7, and it shows that the all original hypotheses are true.

Next, the goodness of fit test is performed on the structural equation model. Table 4 shows the fitting index of the research model in this article. The fitting priority index $(X^{2}/\mathrm{df}$), the normalized fitting index, the Tucker-lewis index, and the comparison fitting index et al., all meet the fitting standard, and the fitting evaluation is ideal. Although the values of the root-mean-square error approximation and the goodness of fitting index are not extremely ideal, they are within a reasonable range. Moreover, the linear regression coefficient is very significant, indicating that the model is well fitted.

**Table 4:** The fitting index of the research model.

$X^{2}/df$	RFI	RMSEA	GFI	IFI	NFI	TLI	CFI
4.403	0.901	0.086	0.816	0.932	0.912	0.923	0.932

**Table 5:** The data of common method deviation test (IEE is dependent variable).

Variables	B	SE	T	Sig.	1/VIF	VIF
IEE	−0.016	0.126	−0.129	0.898		
Entrepreneurial intention	0.149	0.035	4.264	0.000	0.304	3.287
Innovation behavior	0.093	0.046	2.020	0.044	0.210	4.768
Entrepreneurial knowledge	0.864	0.035	24.634	0.000	0.379	2.638
Entrepreneurial attitude	−0.082	0.041	−2.005	0.046	0.373	2.682

### Common Method Deviation Test

Although the questionnaire has been revised repeatedly in the pre-survey process of this study, the problem of identity of data sources cannot be completely avoided. So, it is necessary to further verify the existence of common method deviation. The test results show that the values of variance inflation factor are all less than 10 and the values of tolerability between 5 variables are all more than 0.2. Therefore, this study does not suffer from serious common method bias and collinearity problems.

### Hypothesis Testing

#### Correlation Analysis

In order to acquire the relationship between entrepreneurship education, entrepreneurial knowledge, entrepreneurial attitude, innovative behavior, and entrepreneurial intention, this study conducted correlation analysis on the variables described above. The results are shown in Table 6. As can be seen, gender (β=0.004) has no significant correlation with entrepreneurial knowledge. And education period (β=0.116, *p*<0.05) has significant correlation with innovative behavior. The result indicates that the innovative behavior of college students will vary with the different education phase. The higher the education level is, the higher the innovative behavior is. Meanwhile, the results show that entrepreneurship education, entrepreneurial knowledge, entrepreneurial attitude, innovative behavior, and entrepreneurial intention are positively correlated. Especially, entrepreneurial knowledge had the most significant correlation with innovation and entrepreneurship education (β=0.907, *p*<0.01). However, whether the hypothesis proposed in this study is valid needs to be further analyzed by establishing a regression model.

**Table 6:** Correlation analysis.

Variables	1	2	3	4	5	6	7	8
Educational level	1							
Major	−0.068	1						
der	−0.065	−0.055	1					
IEE	0.050	−0.043	0.012	1				
Entrepreneurial knowledge	0.095	−0.039	0.004	0.907^**^	1			
Entrepreneurial attitude	0.087	−0.011	0.065	0.664^**^	0.713^**^	1		
Innovation behavior	0.116^*^	−0.041	−0.023	0.759^**^	0.758^**^	0.762^**^	1	
Entrepreneurial intention intention	0.073	−0.047	−0.066	0.706^**^	0.670^**^	0.675^**^	0.830^**^	1

* means at the significant level of 0.05;

** means at the significant level of 0.01.

#### Regression Analysis

In order to further explore the relationship between the variables above, the authors adopt multiple regression analysis method to analyze the obtained data. We construct 10 regression models to test the hypothesis, taking education stage, major and gender as control variables. Results of the regression analysis of the above independent variables and entrepreneurial intention are shown in Table 7. The regression models are as follows: innovation and entrepreneurship education (Model 1), entrepreneurial knowledge (Model 2), innovative behavior (Model 3), and entrepreneurial attitude (Model 4).

**Table 7:** Regression analysis results (dependent variable: Entrepreneurial intention).

	Model 1	Model 2	Model 3	Model 4
IEE	0.716^**^			
Entrepreneurial knowledge		0.748^**^		
Innovation behavior			0.910^**^	
Entrepreneurial attitude				0.878^**^
Educational level	0.057	0.006	−0.051	0.005
Major	−0.018	−0.024	−0.017	−0.045
Gender	−0.140	−0.134	−0.094	−0.215
R^2^	0.506	0.454	0.693	0.470
Adj-R^2^	0.501	0.449	0.690	0.465
F	104.624	80.628	230.594	90.807

** means at the significant level of 0.01; ^*^means at the significant level of 0.05.

As shown in Table 7, IEE effectively explained 50.1% of the variation in entrepreneurial intention, and the positive correlation was significant (β=0.716, *p*<0.01). It indicates that IEE has a positive effect on entrepreneurial intention. Therefore, the hypothesis “H1: IEE has a positive impact on entrepreneurial intention” is true. Entrepreneurial knowledge effectively explained 44.9% of the variation of entrepreneurial intention, and there was a significant positive correlation (β=0.748, *p*<0.01). Thus, hypothesis H2 is established. Innovation behavior effectively explained 69% of the variation of entrepreneurial intention, and the positive correlation was significant (β=0.910, p<0.01), showing a very significant positive correlation between innovation behavior and entrepreneurial intention, and hypothesis H3 is established. Entrepreneurial attitude effectively explained 46.5% of the variation of entrepreneurial intention, and there was a significant positive correlation (β=0.878, p<0.01). It indicates that entrepreneurial attitude has a positive effect on entrepreneurial intention, and so, hypothesis H4 is true.

The regression results of IEE and entrepreneurial knowledge (Model 5), innovative behavior (Model 6), and entrepreneurial attitude (Model 7) are shown in Table 8. IEE effectively explained 82.3% of entrepreneurial knowledge, and the positive correlation is significant (β=0.822, p<0.01). It indicates that IEE has a positive effect on entrepreneurial knowledge. Hypothesis H5 is established. IEE effectively explained 57.9% of innovation behavior variation, and there is a significant positive correlation between the two (β=0.702, p<0.01). It indicates that IEE has a positive effect on innovation behavior. Hypothesis H6 is valid. IEE effectively explain 44.3% of entrepreneurial attitude, and there is a significant positive correlation (β=0.522, p<0.01). It indicates that entrepreneurial attitude has a positive effect on entrepreneurial intention. Hypothesis H7 is true.

**Table 8:** Regression analysis results **(**independent variable: IEE**).**

	Model 5	Model 6	Model 7
Dependent variable	Entrepreneurial knowledge	Innovative behavior	Entrepreneurial attitude
Educational level	0.081	0.127	0.085
Major	0.003	−0.004	0.019
Gender	−0.006	−0.048	0.093
β	0.822^**^	0.702^**^	0.522^**^
R^2^	0.825	0.583	0.449
Adj-R^2^	0.823	0.579	0.443
F	481.150	142.910	83.190

** means at the significant level of 0.01; ^*^means at the significant level of 0.05.

The mediating effect results of this paper is shown in Table 9. In Model 8, IEE and entrepreneurial attitude are taken as independent variables to conduct regression analysis with entrepreneurial intention. The results show that the positive effect of innovation and entrepreneurial education is still significant, but the regression coefficient decreases from 0.716 to 0.461, indicating that entrepreneurial attitude plays a partial mediating role between entrepreneurial education and entrepreneurial intention. Therefore, hypothesis H8 is true. Model 9 takes IEE and innovation behavior as independent variables to conduct regression analysis with entrepreneurial intention. The results show that the positive effect of entrepreneurship education is still significant, but the regression coefficient decreases from 0.716 to 0.184. It indicated that the influence is greatly weakened and indicated that innovation behavior plays a partial intermediary role between entrepreneurial education and entrepreneurial intention. Thus, Hypothesis H9 is true. In Model 10, IEE and entrepreneurial knowledge are taken as independent variables to conduct regression analysis with entrepreneurial intention. Moreover, the results show that entrepreneurial education still has a significant positive effect. However, the regression coefficient decreases from 0.716 to 0.573. This suggests that entrepreneurial knowledge plays an intermediary role between entrepreneurial education and entrepreneurial intention.

**Table 9:** Regression analysis results (dependent variable: Entrepreneurial intention**).**

	Model 8	Model 9	Model 10
IEE	0.461^**^	0.184^**^	0.573^**^
Entrepreneurial knowledge			0.175^**^
Innovation behavior		0.759^**^	
Entrepreneurial attitude	0.488^**^		
Educational level	0.016	−0.039	0.043
Major	−0.028	−0.015	−0.019
Gender	−0.185	−0.103	−0.139
R^2^	0.585	0.707	0.510
Adj-R^2^	0.580	0.703	0.504
F	114.997	196.474	84.934

** means at the significant level of 0.01; ^*^means at the significant level of 0.05.

### Robustness Test

We used family background as a newly added independent variable to perform robustness tests. The regression results are shown in Table 10. The results did not change, indicating that the results of our study were relatively robust.

**Table 10:** Robustness test of add independent variable (dependent variable: Entrepreneurial intention).

	Model 1	Model 2	Model 3	Model 4
IEE	0.710^**^			
Entrepreneurial knowledge		0.740^**^		
Innovation behavior			0.903^**^	
Entrepreneurial attitude				0.870^**^
Educational level	0.048	−0.005	−0.063	−0.014
Major	−0.013	−0.017	−0.010	−0.033
Gender	−0.134	−0.126	−0.086	−0.201
Family background	0.040	0.049	0.053	0.084
R^2^	0.507	0.457	0.696	0.478
Adj-R^2^	0.501	0.450	0.692	0.472
F	84.081	68.605	186.841	78.813

** means at the significant level of 0.01; ^*^means at the significant level of 0.05.

## Conclusion and Expectation

### Conclusion

Based on the assimilation learning theory of cognitive structure and planned behavior theory, we constructed and verified the mediating effect model of the mechanism between IEE and entrepreneurial intention. The conclusion as follows: First, IEE has a positive impact on entrepreneurial intention. Accepting entrepreneurship education and learning knowledge related to entrepreneurship is conducive to enhancing students’ entrepreneurial intention. Second, entrepreneurial attitude, innovative behavior, and entrepreneurial knowledge have a significant positive impact on entrepreneurial intention. Third, entrepreneurship education has a positive impact on entrepreneurial attitude, innovative behavior, and entrepreneurial knowledge. Entrepreneurship education positively influences students’ entrepreneurial attitude. Fourth, entrepreneurial knowledge plays a mediating role between entrepreneurial education and entrepreneurial intention, while entrepreneurial attitude and innovative behavior play a partially mediating role between entrepreneurial education and entrepreneurial intention. This explains the influence path of entrepreneurial education on entrepreneurial intention.

### Theoretical Contribution

Generally speaking, the study has made important contributions to the literature on innovation and entrepreneurship education especially from the perspective of educational psychology.

Firstly, this paper is a useful supplement to the literature on innovation and entrepreneurship education. Existing literature suggests that there is a positive relationship between entrepreneurial education and entrepreneurial intention (Packham et al., 2010; Mushtaq et al., 2011). This article empirically tested that innovation and entrepreneurship education can improve the entrepreneurial intention of college students and provided new empirical evidence for the promotion of innovation and entrepreneurship education in universities.

Secondly, this paper expands the research scope of innovation and entrepreneurship. Isidora Ljumović consider that encouraging entrepreneurial culture and developing entrepreneurial education are key factors for the development of modern economies and society as a whole (Ljumović et al., 2019). Some studies have found that most excellent entrepreneurs have strong entrepreneurial intentions in college, and some studies have found that appropriate innovation and entrepreneurship education can significantly improve the entrepreneurial intentions of college students (Vukmirović, 2019). However, few scholars have studied the mechanism between innovation and entrepreneurship education and entrepreneurial intention of college students. This paper measures the concept of innovation and entrepreneurship education, establishes a model with entrepreneurial knowledge, entrepreneurial attitude, and innovative behavior as the intermediary variables, and systematically tests the role of innovation and entrepreneurship education in improving the entrepreneurial intention of college students.

Thirdly, this paper puts forward a new theoretical explanation for China’s innovation and entrepreneurship education from the perspective of educational psychology. Existing studies show that innovation and entrepreneurship education significantly enhances the entrepreneurial intention of college students (Palalic et al., 2016; Halvari et al., 2019). The research in this paper verifies and supports this conclusion from the background of planned behavior theory and cognitive structure assimilation learning theory, that is, innovation and entrepreneurship education has a positive role in promoting the creative intention of college students. Also practical and educational implications are discussed.

### Suggestions

According to the above research conclusions, the effectiveness of IEE in universities can be improved from the following aspects to enhance the entrepreneurial intention of students. First, a sound IEE mechanism should be established. IEE is particularly important in enlightening students’ entrepreneurial intention. It can connect students with innovation and entrepreneurship and inspire innovative and entrepreneurial ideas, which are helpful to promote the formation of entrepreneurial intention. Second, colleges and universities should optimize the innovation and entrepreneurship course system and improve students’ knowledge structure of entrepreneurship. From the empirical data, it can be seen that the imparting of entrepreneurial knowledge has a significant impact on the entrepreneurial intention of college students. Therefore, it is necessary to set up a curriculum system, build entrepreneurial incubation bases, enrich students’ entrepreneurial knowledge, improve their entrepreneurial practical ability, and promote the formation of students’ entrepreneurial intention. Third, a positive entrepreneurial culture should be fostered. Entrepreneurial culture is the sum of values, realistic conditions, characteristics, and social environment that promote individual entrepreneurial behavior. Institution of higher education should gradually form a good atmosphere of encouraging innovation and tolerating failure, exert a positive influence on the entrepreneurial attitude and innovative behavior of college students imperceptibly and silently, and promote college students to form a general consensus of daring to try and explore.

### Research Limitations and Future Prospects

First, this work conducted a survey in the form of questionnaire and collected static cross-sectional data. Dynamically track the entrepreneurial willingness of the survey samples is lack. So, dynamic tracking survey sample is a starting point of research perspective in the future. Second, college students’ understanding of innovation varies with different majors, so it is impossible to fully guarantee the consistency of the research objects’ understanding of innovation behaviors. Different self-evaluation abilities will lead to errors in the evaluation of individuals’ attitude toward entrepreneurship. Finally, in order to further expand the research path, future studies can explore whether there are moderating variables in the formation of this mechanism, such as learning style, the mechanism of action of other factors can be considered to consolidate the results of this study.

## Data Availability Statement

The raw data supporting the conclusions of this article will be made available by the authors, without undue reservation.

## Ethics Statement

The studies involving human participants were reviewed and approved by Hefei University of Technology Ethics Committee. The patients/participants provided their written informed consent to participate in this study. Written informed consent was obtained from the individual(s) for the publication of any potentially identifiable images or data included in this article.

## Author Contributions

All authors listed have made a substantial, direct and intellectual contribution to the work, and approved it for publication.

## Funding

This research was supported by the fund (Grant No. 2020xhx074). This research was supported by the fund (Grant No. szxy2020ccjy02).

## Conflict of Interest

The authors declare that the research was conducted in the absence of any commercial or financial relationships that could be construed as a potential conflict of interest.

## Publisher’s Note

All claims expressed in this article are solely those of the authors and do not necessarily represent those of their affiliated organizations, or those of the publisher, the editors and the reviewers. Any product that may be evaluated in this article, or claim that may be made by its manufacturer, is not guaranteed or endorsed by the publisher.
